# Enzyme-Aided Extraction of Fucoidan by AMG Augments the Functionality of EPCs through Regulation of the AKT/Rheb Signaling Pathway

**DOI:** 10.3390/md17070392

**Published:** 2019-07-03

**Authors:** Vinoth Kumar Rethineswaran, Yeon-Ju Kim, Woong Bi Jang, Seung Taek Ji, Songhwa Kang, Da Yeon Kim, Ji Hye Park, Le Thi Hong Van, Ly Thanh Truong Giang, Jong Seong Ha, Jisoo Yun, Dong Hyung Lee, Sun-Nyoung Yu, Sul-Gi Park, Soon-Cheol Ahn, Sang-Mo Kwon

**Affiliations:** 1 Convergence Stem Cell Research Center, Pusan National University, Yangsan 50612, Korea; 2 Laboratory for Vascular Medicine and Stem Cell Biology, Department of Physiology, School of Medicine, Pusan National University, Yangsan 50612, Korea; 3 Department of Obstetrics and Gynecology, Biomedical Research Institute, Pusan National University School of Medicine, Busan 46241, Korea; 4 Department of Microbiology and Immunology, Pusan National University School of Medicine, Yangsan 50612, Korea; 5 Research Institute of Convergence Biomedical Science and Technology, Pusan National University Yangsan Hospital, Yangsan 50612, Korea

**Keywords:** endothelial progenitor cells, cell proliferation, fucoidan, amyloglucosidase, vascular regeneration

## Abstract

The purpose of the present study is to improve the endothelial progenitor cells (EPC) activation, proliferation, and angiogenesis using enzyme-aided extraction of fucoidan by amyloglucosidase (EAEF-AMG). Enzyme-aided extraction of fucoidan by AMG (EAEF-AMG) significantly increased EPC proliferation by reducing the reactive oxygen species (ROS) and decreasing apoptosis. Notably, EAEF-AMG treated EPCs repressed the colocalization of TSC2/LAMP1 and promoted perinuclear localization of mTOR/LAMP1 and mTOR/Rheb. Moreover, EAEF-AMG enhanced EPC functionalities, including tube formation, cell migration, and wound healing via regulation of AKT/Rheb signaling. Our data provided cell priming protocols to enhance therapeutic applications of EPCs using bioactive compounds for the treatment of CVD.

## 1. Introduction

Cardiovascular disease (CVD) is a leading cause of morbidity and mortality throughout the world and is related to the improper growth and functioning of blood vessels [1,2]. Several factors contribute to CVD, including age, diabetes, ischemia, and atherosclerosis. Various therapeutic approaches have attempted to protect the heart from the progression of CVD, including microRNAs and stem cell therapy. The therapeutic applications of stem cells, including cardiac progenitor cells (CPC), EPCs, and embryonic stem cells (ESCc), receive a disproportionate amount of researcher’s attention.

Angiogenesis is the formation of new blood vessels from existing vasculature through sprouting and intussusception. EPCs isolated from circulating blood, bone marrow, and umbilical cords have the ability to form new blood vessels [3,4]. During tissue or vascular injury hematopoietic stem cells (HSCs) or EPCs migrate and differentiate into endothelial cells and are associated with vascular regeneration and repair [5,6]. Diabetes, ischemia, atherosclerosis impaired redox homeostasis, and generation of reactive oxygen species (ROS), lead to EPC dysfunction, followed by diminished mobilization or depletion of EPCs, which contributes to CVD development [7,8]. Our results were consistent with the findings of previous studies that found transplantation of healthy EPCs replaced damaged cells and repaired injured tissues of hind limb ischemia model [9]. Therefore, EPC-based treatments could be considered for cardiovascular repair and regeneration. Therapeutic application of EPCs is inadequate due to low quality, and quantity, of research. Overcoming these issues and improving EPC survival and functionality is essential. 

Fucoidan, a sulfated polysaccharide found in brown algae and seaweed displays a variety of biological activities, including anti-senescence, anti-apoptotic, anti-tumor, anti-inflammatory, and anti-oxidative functions. Fucoidan and other pharmacological agents steadily improved EPCs bioavailability and increased cell numbers and their functional properties [10]. Moreover, fucoidan reverses senescence of endothelial colony-forming cells (ECFCs). Transplantation of sulphated polysaccharide fucoidan-treated cells rescue ischemic mice model, through improving blood perfusions [9]. Hence, fucoidan-based cell priming protocols might provide therapeutic strategies for the usage and development of EPCs in treating CVD. 

AKT, also known as protein kinase B (PKB) signaling is a key modulator of EPC homing, cellular trafficking, angiogenesis and deregulation of AKT signaling is implicated in many human diseases [11]. AKT activates and translocates the downstream target tuberous sclerosis complex (TSC1-TSC2-TBDC17) from the nucleus to cytosol, or directly inhibits the TSC complex. TSC is a tumor-suppressing gene which inhibits cell growth by acting as a GTPase-activating protein toward Rheb [12,13]. TSC complex directly or indirectly controls Rheb, which subsequently activates mechanistic target of rapamycin complex 1 (mTORC1) [12,14]. Hyper-activation of mTOR has been implicated in various disorders, including diabetes, metabolic syndrome, and angiogenic defect. Substantial evidence supports the importance of AKT/mTOR signaling in cell growth, proliferation, differentiation, and angiogenesis of EPCs [15,16]. AKT/mTOR signaling has attracted interest for its ability to improve EPCs ability to halt CVD progression.

The aim of this study is to develop an effective bioactive compound from fucoidan using novel enzyme-aided extraction by amyloglucosidase (EAEF-AMG), and further explore the beneficial effects of EPCs molecular mechanisms and angiogenic properties.

## 2. Results

### 2.1. Development of Enzyme-Aided Extraction Protocols of Fucoidan 

**Figure 1:**
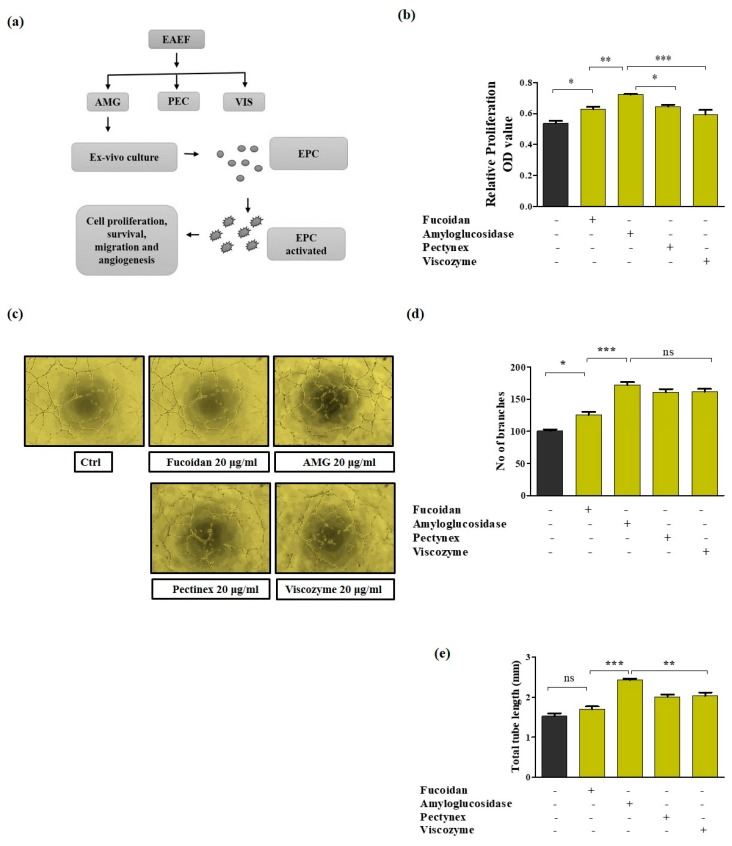
Enzyme-aided extraction of fucoidan enhances EPCs proliferation and tube formation. (**a**) Proposed experimental design. (**b**) Proliferation rate of the EPC cells treated with enzyme-aided extraction of fucoidan treated with equivalent concentration (20 μg/mL) of AMG, viscozyme, or pectinex for 24 h assessed using WTS-1. (**c**–**e**) Tube formation assay was performed using matrigel coated plates for 6 h, then capillary structure was visualized using a light microscopy (Olympus, Tokyo, Japan), total tube length and branches were quantified using ImageJ software (NIH, Bethesda, MD, USA). Data are presented as mean ± standard error of the mean (SEM). The results are considered statistically significant at * *p* < 0.05; ** *p* < 0.01; *** *p* < 0.001 when compared to untreated groups.

The purpose of this study was to develop the most potent bioactive compound from fucoidan by enzyme-aided extraction and to evaluate it activity in maintenance of endothelial progenitor cells. Fucoidan was hydrolyzed using three different enzymes: AMG, viscozyme, and pectinex. EPCs treated with equivalent concentration of enzyme treated fucoidan (20 µg/mL): AMG, viscozyme, and pectinex, and observed that only the enzyme-aided extraction of fucoidan by AMG (EAEF-AMG) significantly improved cell proliferation in comparison to extraction with viscozyme and pectinex (Figure 1b). To ensure endothelial function, we assessed the tube formation capabilities of EPCs, after treatment with equal concentrations of fucoidan and 20 µg/mL of its enzyme aided extracts: AMG, viscozyme, and pectinex for six hours. EAEF-AMG showed stronger endothelial activities, through increased total tube length and number of branches, than fucoidan and its other enzyme aided extracts viscozyme, and pectinex (Figure 1c–e). Hence, EAEF-AMG was chosen for further evaluation.

### 2.2. Priming of EAEF-AMG Protects EPCs Bioactivities from ROS Stress and Cell Apoptosis

**Figure 2:**
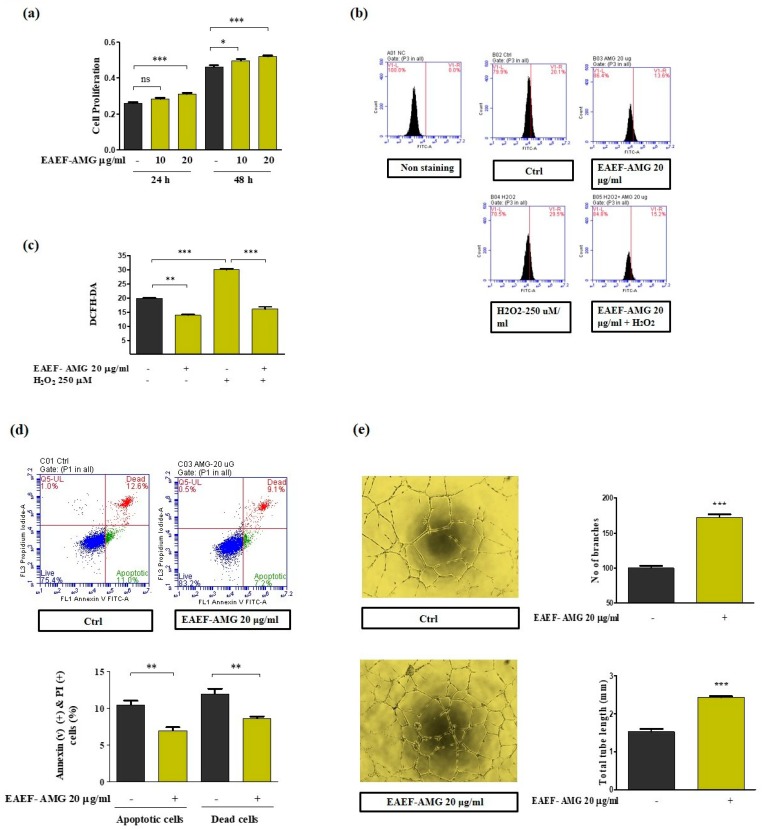

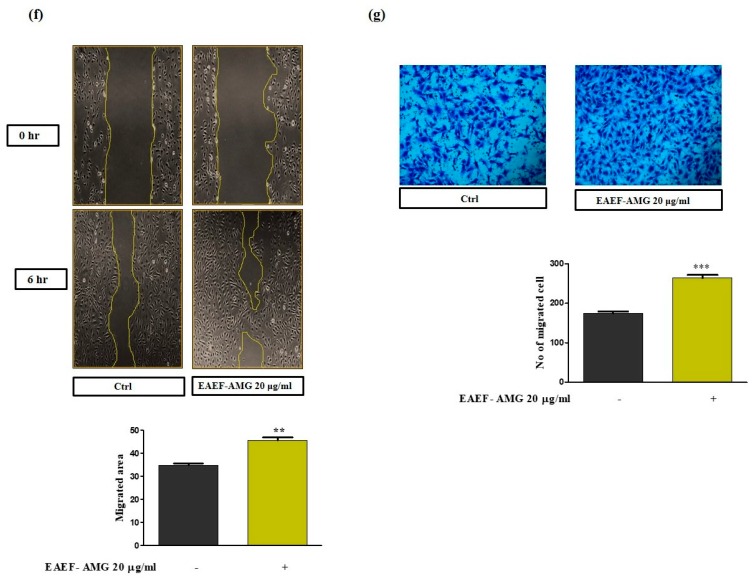
EAEF-AMG mitigates apoptotic cell death and enhances angiogenic activity. (**a**) Cells were treated with EAEF-AMG (10, 20 μg/mL) for 24 and 48 h, then proliferation was measured by WST-1. (**b**,**c**) Carboxy-H2DFFDA was used to measure cellular ROS, cells were pretreated with EAEF-AMG (20 μg/mL) for 24 h, followed by H_2_O_2_ (250 μM) for 5 min, then ROS was measured by FACS. (**d**) Apoptotic cells were measured by Annexin V FITC and propidium iodide staining using FACS. (**e**) For tube formation assay, cells were treated with EAEF-AMG (20 μg/mL) for 6 h, following which capillary structures were visualized using a light microscope (Olympus, Tokyo, Japan). Total tube length and branches were quantified using ImageJ software (NIH, Bethesda, MD, USA). (**f**) Scratch wound healing assays, were performed by cell scratcher for 6 h. Wound healing area was measured using ImageJ software (NIH, Bethesda, MD, USA). (**g**) Transwell migration was performed by seeding cells in the upper inserts of the transwell chamber, whereas medium with EAEF-AMG was added to the lower chamber. The cells were incubated for 6 h and the number of migrated cells was counted in three random fields for each filter (magnification, 20x) under a microscope. Data are presented as mean ± standard error of the mean (SEM). The results are considered as statistically significant at * *p* < 0.05; **p < 0.01; ***p < 0.001 when compared to untreated groups.

Excessive production of ROS in endothelial cells causes endothelial dysfunction and plays a critical role in the development of CVD [17,18]. To assess whether EAEF-AMG protects the EPCs from the oxidative stress induced cell death, we treated the EPCs with different concentration of EAEF-AMG and measured ROS levels and proliferation rate (Figure 2a; Supplementary Figure S1a,b). Pretreatment of EPCs with EAEF-AMG (20 µg/mL) for 24 h protected the cells from the oxidative stress-induced apoptosis of H_2_O_2_ (250 µM). Pretreatment significantly reduced the H_2_O_2_ induced reactive oxygen species levels and apoptosis (Figure 2b–d).

### 2.3. Priming of EAEF-AMG Enhances Angiogenic Activity in EPCs

To investigate whether EAEF-AMG increased angiogenic activity, we performed a tube formation assay using GFR reduced Matrigel coated plates. EPCs treated with 20 μg/mL EAEF-AMG or vascular endothelial growth factor (VEGF-20 ng/mL) for 6 h significantly increased total capillary networks and number of branches compared to untreated cells (Figure 2e). Additionally, EAEF-AMG treated cells displayed significantly enhanced tubular networks and branch points, compared to VEGF treated cells, (Supplementary Figure S2a–c). Moreover, 20 µg/mL of EAEF-AMG treatment for 6 h significantly increased the scratch wound healing and Transwell migration (Figure 2f,g). In addition to determining the proangiogenic cytokine expression levels, cells were treated with EAEF-AMG (20 µg/mL) for 6 h along with respective control and the mRNA expression of the proangiogenic cytokines CXCL12 increased and IL-8 were found to be significantly dysregulated in comparison to the untreated cells. (Supplementary Figure S1c).

### 2.4. Priming of EAEF-AMG Enriches Functional EPCs

**Figure 3:**
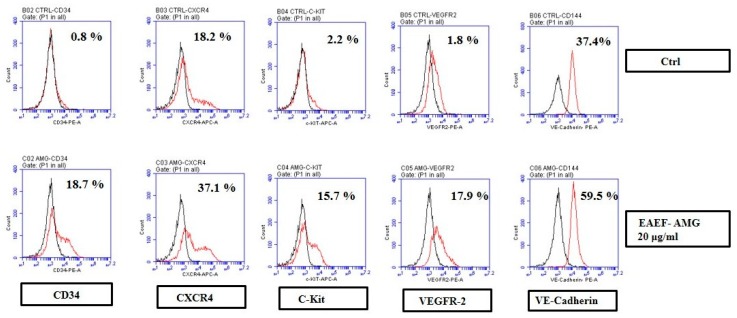
EAEF-AMG enhanced the functional markers expression of EPCs. Cells treated with EAEF-AMG. (20 μg/mL) for 24 h showed increased expression of CD34, CXCR4, C-Kit, VEGFR2, and VE-Cadherin using fluorescence activated cell sorting (FACS). FACS gating was performed using non-stained cells as a negative control. The fraction of positively stained cells was determined by comparison with non-stained cells. The percentage of positively-stained cells is indicated by the positive peaks (red lines indicate cells stained with each antibodies, and black lines indicate the negative control cells).

Endothelial surface markers play a vital role in distinguishing and controlling the cellular function and development. Endothelial cell dysfunction influences the expression of functional markers. To discover whether EAEF-AMG treatment improved the functional marker expression, CD34, C-Kit, CXCR4, VEGFR-2, and VE-cadherin were analyzed by FACS. EPCs treated with 20 μg/mL EAEF-AMG for 24 h, substantially increased the expression of functional markers including CD34, C-Kit, CXCR4, VEGFR-2, and VE-cadherin (Figure 3)

### 2.5. Priming of EAEF-AMG Regulates AKT/Rheb Signaling in EPCs

**Figure 4:**
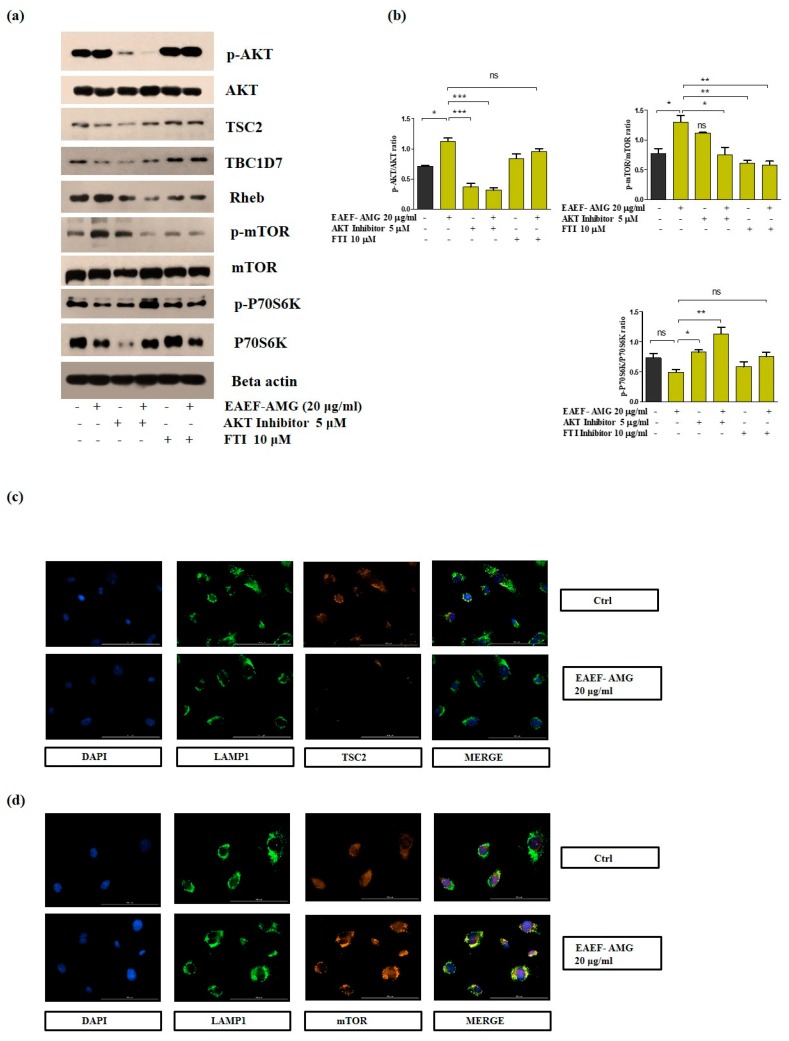
EAEF-AMG regulates the AKT/Rheb signaling pathway. (**a**) Cells were treated with EAEF-AMG (20 μg/mL) and in combination with specific inhibitors such as AKT inhibitor (5 μM), and farnesyltransferase inhibitor (10 μM) for 24 h, then Western blots were performed to evaluate the protein level expression of p-AKT, AKT, TSC2, and TBCD17, Rheb, p-mTOR, mTOR, p-P70S6K, and P70S6K and loading control beta actin. (**b**) Quantification of Western blots. (**c**–**d**) Immunofluorescence was performed by treating cells with EAEF-AMG (20 μg/mL) for 45 min to assess the localization of TSC2/LAMP1 in perinuclear sites, and images of perinuclear aggregation of mTOR/LAMP1 were captured using a 40× objective lens on a Lion Heart FX automated microscope (Biotek, Winooski, VT, USA). Scale bar = 100 μm.

To elucidate the importance of AKT/Rheb signaling in EPCs, Western blotting was performed to analyze the protein expression of AKT and its downstream signaling, including Rheb. Treatment with 20 μg/mL EAEF-AMG for 24 h up-regulated the expression of p-AKT, p-mTOR, and Rheb, subsequently TSC2 and TBDC17, negative regulators of mTOR signaling were down-regulated (Figure 4a,b). We observed that treatment of 20 μg/mL EAEF-AMG for 45 min hindered lysosomal localization of TSC2, a negative regulator of mTOR signaling in comparison to the control (Figure 4c). Additionally, 20 μg/mL EAEF-AMG promoted mTOR/LAMP1 aggregation on perinuclear sites in comparison to the control cells (Figure 4d).

### 2.6. Priming of EAEF-AMG Augments the Functional Activities of EPC via Regulation of AKT/Rheb Signaling

**Figure 5:**
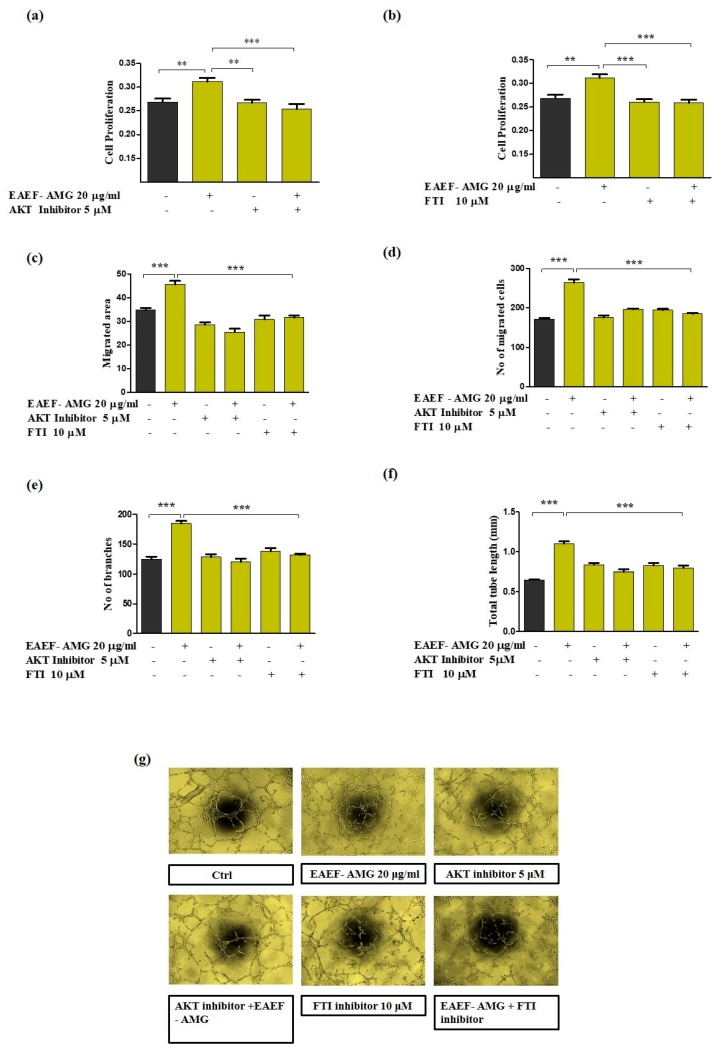
EAEF-AMG enhances angiogenic activity via regulation of AKT/Rheb signaling. (**a**,**b**) Cells treated with EAEF-AMG (20 μg/mL) and in combination with AKT inhibitor (5 μM) and farnesyltransferase inhibitor (10 μM) for 24 h, then proliferation was measured by WST-1. (**c**) Scratch wound healing assay was performed using a cell scratcher and wound healing area was measured after 6 h using ImageJ software (NIH, Bethesda, MD, USA). (**d**) Transwell migration was performed by seeding cells on upper inserts and the lower chamber selectively contained EAEF-AMG, AKT, and farnesyltransferase inhibitor. Next, cells were incubated for 6 h and the number of migrated cells was counted in three random fields per filter (magnification, 20×) using a light microscope. (**e**–**g**) In the tube formation assay, capillary structures were captured after 6 h using a light microscope (Olympus, Tokyo, Japan). Total tube length and branches were quantified using ImageJ software (NIH, Bethesda, MD, USA). Data are presented as mean ± standard error of the mean (SEM). The results are considered as statistically significant at * *p* < 0.05; ** *p* < 0.01; *** *p* < 0.001 when compared to untreated groups.

**Figure 6:**
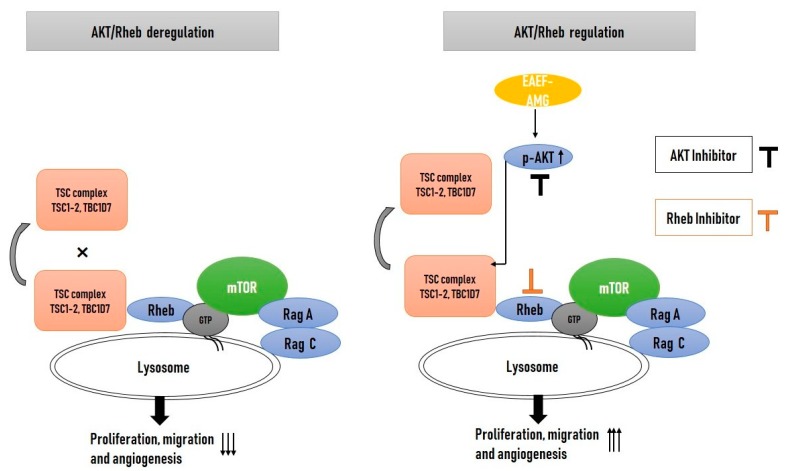
Schematic diagram representation of EAEF-AMG improving angiogenic activity through regulation of AKT/Rheb signaling. Exposure of EAEF-AMG up-regulated the expression of p-AKT, leading to obstruction of the perinuclear accumulation and colocalization of TSC2/LAMP1, followed by augmenting the perinuclear aggregation of mTOR/Lamp1, which further regulates downstream signaling, and intensifies EPC functionality.

To determine whether AKT/Rheb signaling plays a role in EAEF-AMG persuaded increased proliferation, cell migration, wound healing, and tube formation. Treatment of EPC cells with EAEF-AMG and in combination with AKT inhibitor (5 μM) and farnesyltransferase inhibitor (10 μM), inhibitor of Rheb (a direct regulator of mTOR activation) blocked cell proliferation, wound healing, cell migration, and tube formation activity (Figure 5a–g) (Figure 6).

## 3. Discussion

Asahara et al. were the first to isolate EPCs and observed that they had the ability to form new blood vessels [19,20]. EPCs are derived from bone-marrow and released into the blood in response to vascular damage and ischemia [21,22] where they mobilize and differentiate into an endothelial cells are important for vascular regeneration, including vascular formation, remodeling, and repair (wound healing) of blood vessels [23,24]. The number of EPCs, and their migratory activity, are inversely correlated with risk factors for CVD progression [25,26].

Ageing, ischemia, and atherosclerosis impair redox homeostasis in endothelial cells [27]. ROS, when present at optimal levels, play an important physiological role, but excessive production of ROS leads to EPC dysfunction [27] resulting in reduced numbers of EPCs, migration, differentiation, and impaired endothelial function. [28,29]. To expand the therapeutic efficacy of EPCs against CVD progression, EPC survival, proliferation, and endothelial functions must be increased. Previously, we confirmed the role of fucoidan’s inhibiting cellular senescence and augmentation of the angiogenic potential of ECFCs via Integrin-AKT signaling [30]. Our study aimed to restore EPCs, and expand their benefits by using EAEF-AMG for its ability to increase cell proliferation by suppressing ROS and apoptosis (Figure 2a–d). 

EPCs identified and can be distinguished from the other types by the expression of several surface markers, such as CD34, CXCR4, c-KIT, VEGFR-2, and VE-Cadherin, which also further characterize EPCs functional activity such as high proliferative and angiogenic potential [31]. CXCR4 and VEGFR-2 surface expression directs the hematopoietic stem cells, homing, migration, invasion, and angiogenesis and contributes to formation of new blood vessel and repair existing one [4,32,33]. Endothelial cell colony-forming activity increased by c-Kit expression, besides c-Kit deficient mice shows reduction of colony formation [34]. Umbilical cord derived EPCs stimulated by EAEF-AMG, expressed the cell surface markers CD34, CXCR4, c-KIT, VEGFR-2, and VE-Cadherin, increasing endothelial functions, including tube formation, cell migration, and wound healing (Figure 2e–g). In addition, EAEF-AMG more effectively stimulate tubular network and branch number than VEGF treated cells (Supplementary Figure S2).

AKT is a serine/threonine protein kinase with three isoforms. Phosphorylation of AKT inactivates the downstream signaling of TSC complexes through dissociation of TSC2/LAMP1 colocalization in perinuclear sites [12]. We found phosphorylation and activation of AKT (Ser473) by EAEF-AMG increased cell proliferation by hindering the protein level expression of TSC, TBDC17 (Figure 4a and Figure 5a). TSC2/LAMP1 colocalization suppressed the activation of GTPase activating protein of Rheb [12,13], a direct activator of mTOR. We found EAEF-AMG treatment blocked the colocalization of TSC2/LAMP1 (Figure 4c) and regulated downstream protein expression, cell proliferation, wound healing, migration, and capillary formations in an AKT- and Rheb-dependent manner (Figure 6). Inhibition of AKT and Rheb (farnesyltransferase inhibitor) negatively regulates protein expression, cell proliferation, wound healing, migration, and capillary formations (Figure 4 and Figure 5).

Mammalian target sof rapamycin have two distinct complexes, mTORC1 and mTORC2, and their activation can positively influence cellular metabolism and play a crucial role in many cellular functions, including cellular senescence, autophagy, ribosomal biogenesis, protein synthesis, proliferation, differentiation, and angiogenesis [35,36]. Recent studies proposed perinuclear aggregation of mTOR/Rheb and mTOR/LAMP1 promoted mTOR activation [36]. Perinuclear aggregation of mTOR/Rheb and mTOR/LAMP1 with EAEF-AMG treatment was unexpected (Figure 4d) and (Figure 6). Moreover, previous studies demonstrated mTOR hyperactivation increased ribosomal biogenesis and protein synthesis, causing defects in the angiogenesis of endothelial cells [11]. We demonstrated that EAEF-AMG regulated mTOR activation and constrained ribosomal protein synthesis via regulation of AKT/Rheb signaling (Figure 4a).

## 4. Materials and Methods

### 4.1. Isolation of EPCs and Cell Culture

Human umbilical cord blood (HUCB) was collected from healthy volunteers according to the protocol approved by the institutional review board of Pusan National University, Yangsan hospital, South Korea (Approval No. PNUYH-05-2017-053). Mononuclear cells (MNCs) isolated from HUCB by density gradient centrifuge using FIcoll (GE Healthcare, Buckinghamshire, UK). HUCB derived MNCs maintained in an EGM-2 bullet kit system (Lonza, Walkersville, MD, USA) containing endothelial basal medium 2 (EBM-2), 5% fetal bovine serum (FBS), human vascular endothelial growth factor (VEGF), human basic fibroblast growth factor (b-FGF), human epidermal growth factor (EGF), human insulin-like growth factor-1 (IGF-1), ascorbic acid, and GA-1000. Culturing MNCs in EGM-2 media formulations maintain outstanding EPC morphology and function. Cells were seeded on 1% gelatin-coated plates (Sigma, St. Louis, MO, USA) and incubated at 37 °C in 5% CO_2._ After five days, non-adherent cells were discarded and fresh culture medium was added. The adherent cells were assessed for the ability to uptake Dil acetylated-LDL (Thermo Fisher Scientific, Carlsbad, CA, USA) or binding of fluorescently-labeled Ulex- europaeus agglutin 1 plant lectin (Sigma, St. Louis, MO, USA). The adherent cells that uptake of Dil acetylated-LDL or bound Ulex- europaeus agglutin 1 plant lectin were considered EPCs (Supplementary Figure S3). These adherent cells further confirmed by expression of EPCs surface markers by flow cytometry. Cultures were maintained for 10–14 days until the development of colonies. The culture medium was changed daily and colonies were re-plated and cultured for further studies. EPCs were used at passages 6–12 in subsequent experiments.

### 4.2. Enzymatic Extraction of Fucoidan

Enzyme-aided extraction of fucoidan was performed as previously reported [37,38]. Fucoidan, a polysaccharide derived from marine algae, has a high molecular weight and is larger than is typical for use in drug applications, so our study used a lower molecular weight of fucoidan enzymatically extracted by hydrolysis using three commercially available carbohydrate degrading enzymes: AMG, pectinex, and viscozyme (NOVO Nordisc, Bagsvaerd, Denmark). The 1% (*v*/*v*) of fucoidan was suspended in distilled water and then treated with 5% (*v*/*v*) of each of the enzymes consecutively, which were bio transformed at 55 °C for two days to inactivate the enzymes. Then samples were clarified by centrifugation (3000× *g*, for 20 min at 4 °C) [39]. The 10 mg/mL sample was diluted in water and stored at 20 °C for future experiment.

### 4.3. Flow Cytometer Analysis

Cultured EPCs were identified and characterized by flow cytometry using hematopoietic stem cell markers CD34, CXCR4, c-Kit, VEGFR2, and VE-Cadherin were purchased from (BD Pharmingen, Franklin Lake, NJ, USA). The flow cytometry analysis was carried out by fluorescence activated cell sorting (FACS; BD FACS canto 2, San Joes, CA, USA). The percentage of stained cells was indicated by the red line peaks.

### 4.4. WST-1 Proliferation Assay

Cell proliferation was detected using D-Plus cell counting kit (CCK-8 - DonginLS, Seoul, Korea) according to the manufacturer’s instructions. Cells were seeded (5 × 10^3^) into 96 well plates. The cells were incubated overnight at 37 °C in a 5% CO_2_ incubator, and then cells were selectively treated with EAEF-AMG and AKT (Sigma, St. Louis, MO, USA), FTI (Sigma, St. Louis, MO, USA) inhibitor for 24 and, 48 h. Next, cells were treated with (10 μL/well) WST-1 for 2 h in the dark at 37 °C in a 5% CO_2_ incubator, the absorbance of each well was measured at 450 nm using a (Tecan XFluor, Mannedorf, Switzerland) micro plate reader. Each experiment was repeated three times.

### 4.5. Carboxy-H2DFFDA Assay

ROS production was evaluated using carboxy-H2DFFDA probes (Life technologies, Carlsbad, CA, USA). Cells were washed twice with PBS and incubated with 10 µM carboxy-H2DFFDA at 37 °C for 30 min in the dark. Next, the cells were collected and analyzed by FACS (BD Biosciences, San Jose, CA, USA). Total cellular ROS levels were expressed as the average DCF fluorescence intensity of the cells.

### 4.6. Annexin V FITC/PI Staining Assay

Flow cytometry was performed to assess apoptosis by FITC Annexin V apoptosis Detection kit (BD Pharmingen, Franklin Lake, NJ, USA) according to manufacturer’s instructions. Samples were processed and the cells were seeded on six well plates and incubated overnight at 37 °C in a 5% CO_2_ incubator, then selectively treated with AMG for 24 h. The stained cells were analyzed by FACS (BD Facscanto II, San Jose, CA, USA). The Annexin V FITC and PI negative cells were viable, the Annexin V FITC and positive cells were apoptotic, and PI positive cells were dead.

### 4.7. Transwell Migration Assay

Cell migration assays were performed using 24 Transwell migration chamber inserts (8 μm pore size; Costar, Columbia, WA, USA). EPCs (2 × 10^4^/100 μL) were seeded on the upper inserts, and lower chambers were filled with medium, either with, or without, EAEF-AMG, then the cells were incubated for 6 h at 37 °C. Next, cells in the upper membrane were removed with a cotton swab, and cells which migrated to the lower surface were fixed with 4% paraformaldehyde (PFA) and stained with 0.5% crystal violet in 25% methanol for 30 min. The number of migrated cells were counted under a microscope (magnification, 200×) by randomly selecting three fields per filter.

### 4.8. Scratch Wound Healing Assay

A wound healing assay was performed. Cells (2 × 10^5^) were seeded on a 6 well plate and cultured until they reached 90% confluence. Cells were scratched using a cell scratcher (SPL Life science, Seoul, Korea), and then washed with PBS twice. Then cells were incubated in full medium, with or without EAF, for 6 h at 37 °C in a CO_2_ incubator. Wound healing area was measured using ImageJ software (NIH, Bethesda, MD, USA). Cell migration activity was calculated as the migration area: ((initial scratch area A_0h_ − final scratch area A_6 h_)/initial scratch area A_0h_) × 100%.

### 4.9. Matrigel Tube Formation Assay

Growth factor reduced matrigel (BD Biosciences, San Jose, CA, USA) was thawed at 4 °C the day before the experiment and then 96 well plates were coated with matrigel (60 μL/well) and polymerized for 30 min at 37 °C. Cells were seeded on matrigel coated wells at a density of 5 × 10^3^ cells/well in the full medium, selectively treated with EAEF-AMG, AKT inhibitor, farnesyl transferase inhibitor, and VEGF for 6 h at 37 °C in a CO_2_ incubator. Next, tube length and branch formation were measured by counting the number of tubes and their length in one microscopic field per well (20× magnification). Recombinant human VEGF (165) were purchased from PeproTech (Rocky Hill, NJ, USA).

### 4.10. Western Blotting

Proteins were extracted from cell lysates using PRO-PREP protein extraction buffer (Intron biotechnology, Seoul, Korea) and protein concentration was determined by BCA assay kit (Thermo scientific, Rockford, IL, USA). Protein samples were separated with SDS-PAGE, and transferred to PVDF membranes (Millipore, Billerica, MA, USA). The membranes were blocked with 5% skim milk and incubated with primary antibodies p-AKT (Ser 473), AKT, p-mTOR (Ser 2448), mTOR, p-P70S6K (Ser 371), P70S6K (Cell Signaling technology, Boston, MA, USA), TSC2, TBDC17, Rheb, beta actin from (Santa Cruz Biotechnology, Dallas, Texas, USA) overnight at 4 °C, followed by incubation with HRP conjugated goat anti-rabbit IgG (Enzo Life Sciences, Farmingdale, New York, NY, USA) and goat anti-mouse IgG (Enzo Life Sciences, Farmingdale, NY, USA) secondary antibody at 25 °C for 1 h and Western blots were enhanced using chemiluminescent detection (Immobilon, Millipore, Burlington, MA, USA).

### 4.11. Immunofluorescence

In preparation for immunofluorescence, cells (2 × 10^4^ cells/mL) were seeded in 1% gelatin coated two well chamber slides, and then incubated overnight. The following day a portion of the cells were treated with EAEF-AMG for 45 min at 37 °C in a CO_2_ incubator. Following treatment, the cells were fixed with 4% PFA for 10 min at 25 °C. Samples were washed and permeabilized with 0.1% Triton x-100 in Phosphate Buffer Saline (PBS) for 5 min, and blocked with 10% normal goat serum (0.3 M glycine) for 1 h. Cells were stained with primary antibodies (0.3 % Triton x-100) TSC2, mTOR, LAMP1 (Santa Cruz Biotechnology, Dallas, TX, USA) overnight at 4 °C in the dark. After four washes in PBS, secondary antibodies (Alexa flour 488, 594, Life technologies, Carlsbad, CA, USA) were incubated for 1 h at room temperature in the dark. After washing four times with PBS, nuclei were stained with DAPI- 1 μg/mL (Sigma, St. Louis, MO, USA), and washed four times with the same buffer. Slides were covered with a coverslip using ProLong anti-fade diamond mountant and images were captured using a 40× objective lens on a Lion Heart FX automated microscope (Biotek, Winooski, VT, USA). Scale bar: 100 μm.

### 4.12. Quantitative Reverse Transcription-Polymerase Chain Reaction

Total RNA was extracted using TRIzol reagent following the manufacturer’s instruction (Ambion, Life Technologies, Carlsbad, CA, USA). The RNA concentration measured by Nanodrop UV spectrophotometer (Thermoscientific, Waltham, MA, USA). The cDNA synthesized using prime script 1st strand cDNA synthesis kit (TAKARA, Japan). The total RNA was reverse transcribed using SYBR green real time PCR mastermix (Roche, Penzberg, Germany) was used for determining the mRNA levels of different genes. The primers were used IL-8 (F) 5′-GTGCAGTTTTGCCAAGGAGT-3′ and (R) 5′-CTCTGCACCCAGTTTTCCTT-3′, CXCL12 (F) 5′-CTACTCAAGTGCCTCCACGA-3′ and (R) 5′-GGACACACCACAGCACAAAC-3′, GAPDH (F) 5′-AACAGCGACACCCACTCCTC-3′, and (R) 5′-CATACCAGGAAATGAGCTTGACAA-3′. The Roche Light Cycler 96 real-time PCR machine was used for thermal cycling. The data were calculated using double delta Ct analysis and were normalized against control gene (GAPDH).

### 4.13. Statistical Analysis

Statistical analyses were performed using GraphPad Prism software (Version 5—San Diego, CA, USA) and a one-way analysis of variance (ANOVA) was used to assess the differences between experimental and control groups. Data are presented as mean ± standard error of the mean (SEM). The results were considered as statistically significant at *p* < 0.05 (*). *p* values less than 0.01 or 0.001 were indicated with ** or ***, respectively. The experimental data presented was an average of three independent experiments.

## 5. Conclusions

Our study clearly indicated that a short-term EPC priming protocol using EAEF-AMG is a promising therapeutic strategy to promote EPC functions via regulation of the AKT/Rheb signaling pathways.

## Supplementary Materials

The following are available online at https://www.mdpi.com/1660-3397/17/7/392/s1, Figure S1: Enzyme aided extraction of fucoidan by AMG enhances EPC proliferation. (a-b) Enzyme aided extraction of fucoidan by AMG treated with different doses (20, 40, 60, and 80 μg/mL) for 24 h and 48 h, following which cell proliferation was measured by WST-1. (c) RNA was isolated and relative mRNA levels of CXCL12 and IL-8 between control and 20 μg/mL EAEF-AMG treated cells were determined by qRT-PCR. Data are presented as mean ± standard error of the mean (SEM). The results are considered as statistically significant at * *p* < 0.05; ** *p* < 0.01; *** *p* < 0.001 when compared to untreated groups. Figure S2: EAEF-AMG and VEGF enhances angiogenic activity. (a) Cells were treated with EAEF-AMG (20 μg/mL) or VEGF (20 ng/mL) for 6 h, and then capillary structures were acquired using a light microscope (Olympus, Tokyo, Japan). Total tube length and branches were quantified using ImageJ software (NIH, Maryland, USA). Data are presented as mean ± standard error of the mean (SEM). The results are considered as statistically significant at * *p* < 0.05; ** *p* < 0.01; *** *p* < 0.001 when compared to untreated groups. Figure S3: Characterization of EPCs. EPCs phenotype was confirmed by immunofluorescence, by uptake of Dil acetylated-LDL or binding of fluorescently labeled Ulex- europaeus agglutin 1 plant lectin.
