# Supplementary material for: Enzyme-Aided Extraction of Fucoidan by AMG Augments the Functionality of EPCs through Regulation of the AKT/Rheb Signaling Pathway
Source: Mar Drugs. 2019 Jul 3;17(7):392. doi: 10.3390/md17070392 (PMC6669526; doi:10.3390/md17070392)
Supplement: Supplementary file 1 [file marinedrugs-17-00392-s001.pdf]

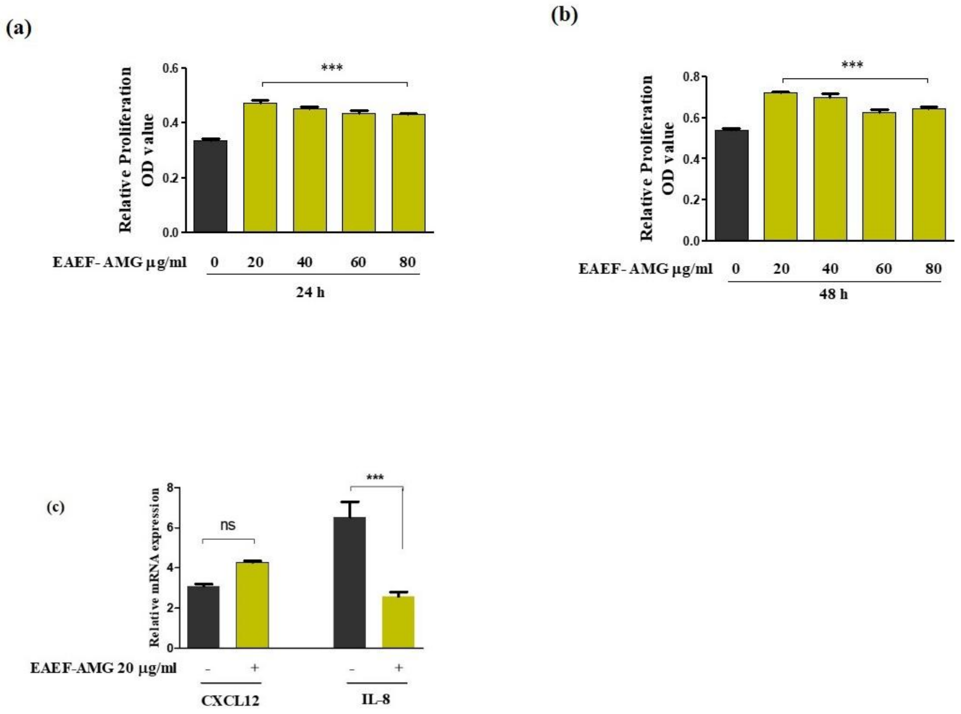

Supplementary Figure 1. Enzyme aided extraction of fucoidan by AMG enhances EPC proliferation. (a-b) Enzyme aided extraction of fucoidan by AMG treated with different doses (20, 40, 60, and 80 µg/mL) for 24 h and 48 h, following which cell proliferation was measured by WST-1. (c) RNA was isolated and relative mRNA levels of CXCL12 and IL-8 between control and 20 µg/mL EAEF-AMG treated cells were determined by qRT-PCR. Data are presented as mean ± standard error of the mean (SEM). The results are considered as statistically significant at *P < 0.05; **P < 0.01; ***P < 0.001 when compared to untreated groups.

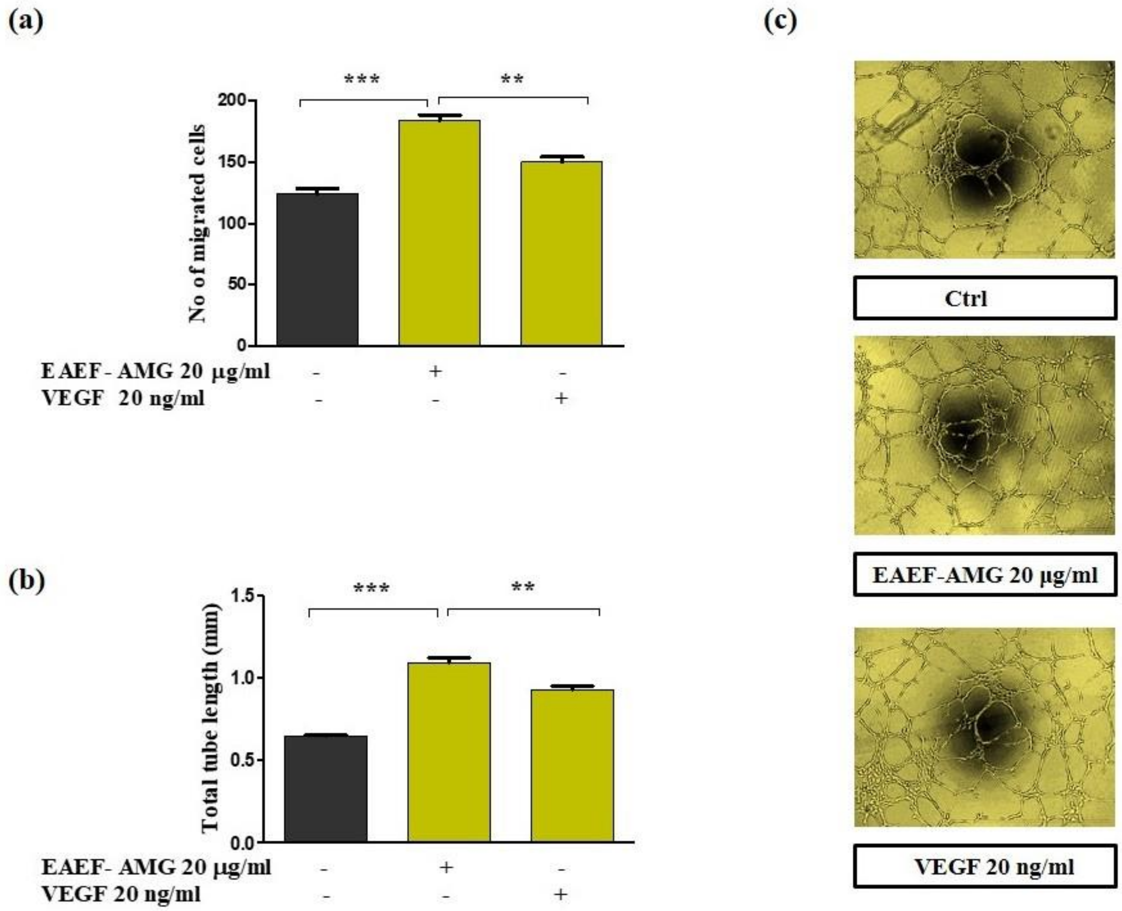

Supplementary Figure 2. EAEF-AMG and VEGF enhances angiogenic activity. (a) Cells were treated with EAEF-AMG (20 μg/mL) or VEGF (20 ng/mL) for 6 h, and then capillary structures were acquired using a light microscope (Olympus, Tokyo, Japan). Total tube length and branches were quantified using ImageJ software (NIH, Maryland, USA). Data are presented as mean ± standard error of the mean (SEM). The results are considered as statistically significant at *P < 0.05; **P < 0.01; ***P < 0.001 when compared to untreated groups.

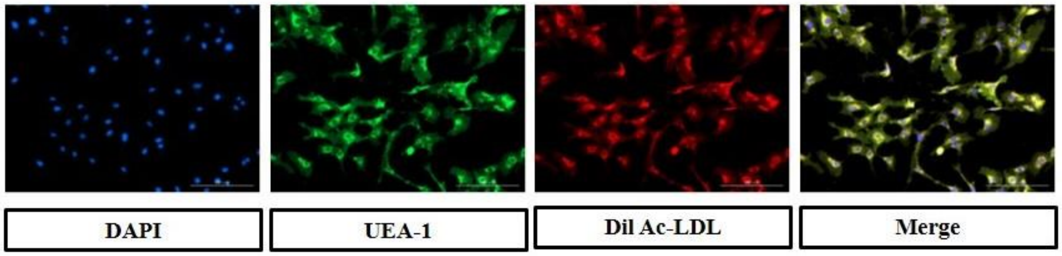

Supplementary Figure 3. Characterization of EPCs. EPCs phenotype was confirmed by immunofluorescence, by uptake of Dil acetylated-LDL or binding of fluorescently labeled Ulex- europaeus agglutin 1 plant lectin
